# Supplementary material for: Multi‐dataset identification of innovative feature genes and molecular mechanisms in keratoconus
Source: J Cell Mol Med. 2024 Sep 19;28(18):e70079. doi: 10.1111/jcmm.70079 (PMC11412914; doi:10.1111/jcmm.70079)
Supplement: Supplementary file 2 — Material S2. [file JCMM-28-e70079-s002.zip › 7.GSEA analysis/5.RNF217-AS1/KEGG_MELANOGENESIS.html]

Details for gene set KEGG\_MELANOGENESIS[GSEA]

|  || Dataset | RNF217-AS1.group.cls#h\_versus\_l |
| Phenotype | group.cls#h\_versus\_l |
| Upregulated in class | l |
| GeneSet | KEGG\_MELANOGENESIS |
| Enrichment Score (ES) | -0.4347139 |
| Normalized Enrichment Score (NES) | -1.6399558 |
| Nominal p-value | 0.006147541 |
| FDR q-value | 0.32699084 |
| FWER p-Value | 0.532 |
Table: GSEA Results Summary

  

Fig 1: Enrichment plot: KEGG\_MELANOGENESIS      
 Profile of the Running ES Score & Positions of GeneSet Members on the Rank Ordered List

  

| SYMBOL | TITLE | RANK IN GENE LIST | RANK METRIC SCORE | RUNNING ES | CORE ENRICHMENT || 1 | PRKCG | na | 203 | 0.657 | 0.0348 | No |
| 2 | CALML6 | na | 1514 | 0.391 | 0.0211 | No |
| 3 | WNT9A | na | 2856 | 0.297 | 0.0008 | No |
| 4 | ADCY2 | na | 2970 | 0.293 | 0.0156 | No |
| 5 | CAMK2A | na | 3603 | 0.267 | 0.0139 | No |
| 6 | ADCY4 | na | 4384 | 0.238 | 0.0061 | No |
| 7 | WNT9B | na | 4560 | 0.231 | 0.0154 | No |
| 8 | ADCY5 | na | 4777 | 0.225 | 0.0230 | No |
| 9 | CAMK2B | na | 5000 | 0.217 | 0.0300 | No |
| 10 | ADCY7 | na | 5579 | 0.197 | 0.0255 | No |
| 11 | WNT8A | na | 5716 | 0.192 | 0.0334 | No |
| 12 | MC1R | na | 5728 | 0.192 | 0.0450 | No |
| 13 | GNAQ | na | 7033 | 0.152 | 0.0167 | No |
| 14 | PLCB3 | na | 7444 | 0.141 | 0.0136 | No |
| 15 | GNAI3 | na | 7550 | 0.139 | 0.0192 | No |
| 16 | GNAO1 | na | 9047 | 0.104 | -0.0176 | No |
| 17 | WNT3 | na | 9757 | 0.090 | -0.0325 | No |
| 18 | ADCY1 | na | 10624 | 0.076 | -0.0528 | No |
| 19 | GSK3B | na | 10962 | 0.071 | -0.0582 | No |
| 20 | WNT2 | na | 11310 | 0.066 | -0.0641 | No |
| 21 | KRAS | na | 11445 | 0.064 | -0.0641 | No |
| 22 | CREB3 | na | 11909 | 0.057 | -0.0739 | No |
| 23 | FZD9 | na | 12064 | 0.055 | -0.0750 | No |
| 24 | TCF7L2 | na | 12940 | 0.043 | -0.0976 | No |
| 25 | MAPK1 | na | 13362 | 0.038 | -0.1074 | No |
| 26 | CALM2 | na | 13884 | 0.032 | -0.1205 | No |
| 27 | MAPK3 | na | 14127 | 0.029 | -0.1257 | No |
| 28 | DVL1 | na | 14350 | 0.026 | -0.1305 | No |
| 29 | CREBBP | na | 14557 | 0.024 | -0.1349 | No |
| 30 | WNT3A | na | 14964 | 0.019 | -0.1454 | No |
| 31 | GNAI2 | na | 14991 | 0.019 | -0.1450 | No |
| 32 | CALML5 | na | 15459 | 0.014 | -0.1576 | No |
| 33 | DVL3 | na | 15800 | 0.011 | -0.1668 | No |
| 34 | HRAS | na | 15878 | 0.010 | -0.1684 | No |
| 35 | CAMK2G | na | 16021 | 0.008 | -0.1719 | No |
| 36 | MAP2K2 | na | 16236 | 0.006 | -0.1777 | No |
| 37 | WNT4 | na | 16250 | 0.006 | -0.1777 | No |
| 38 | CREB1 | na | 16423 | 0.004 | -0.1824 | No |
| 39 | TCF7L1 | na | 16424 | 0.004 | -0.1821 | No |
| 40 | CREB3L3 | na | 16617 | 0.003 | -0.1875 | No |
| 41 | ADCY9 | na | 17206 | -0.004 | -0.2043 | No |
| 42 | NRAS | na | 17304 | -0.005 | -0.2068 | No |
| 43 | CALML3 | na | 17310 | -0.005 | -0.2066 | No |
| 44 | WNT1 | na | 17572 | -0.007 | -0.2137 | No |
| 45 | GNAS | na | 17807 | -0.009 | -0.2199 | No |
| 46 | CALM3 | na | 18318 | -0.014 | -0.2338 | No |
| 47 | FZD5 | na | 18663 | -0.017 | -0.2427 | No |
| 48 | RAF1 | na | 18969 | -0.020 | -0.2503 | No |
| 49 | ADCY6 | na | 19355 | -0.024 | -0.2599 | No |
| 50 | WNT10A | na | 19364 | -0.024 | -0.2587 | No |
| 51 | FZD6 | na | 19485 | -0.025 | -0.2606 | No |
| 52 | CTNNB1 | na | 19818 | -0.028 | -0.2684 | No |
| 53 | TCF7 | na | 19939 | -0.029 | -0.2701 | No |
| 54 | WNT7B | na | 19942 | -0.029 | -0.2683 | No |
| 55 | CREB3L2 | na | 20073 | -0.031 | -0.2702 | No |
| 56 | CAMK2D | na | 20347 | -0.033 | -0.2760 | No |
| 57 | CREB3L1 | na | 20661 | -0.036 | -0.2828 | No |
| 58 | EDN1 | na | 21356 | -0.044 | -0.3001 | No |
| 59 | PLCB4 | na | 21374 | -0.044 | -0.2979 | No |
| 60 | CREB3L4 | na | 21836 | -0.049 | -0.3082 | No |
| 61 | WNT7A | na | 21895 | -0.050 | -0.3068 | No |
| 62 | ADCY8 | na | 21903 | -0.050 | -0.3039 | No |
| 63 | ADCY3 | na | 22794 | -0.059 | -0.3260 | No |
| 64 | PRKACA | na | 23066 | -0.063 | -0.3299 | No |
| 65 | MAP2K1 | na | 23215 | -0.064 | -0.3302 | No |
| 66 | EP300 | na | 23570 | -0.069 | -0.3362 | No |
| 67 | WNT10B | na | 23653 | -0.070 | -0.3342 | No |
| 68 | PRKCA | na | 23805 | -0.072 | -0.3341 | No |
| 69 | WNT2B | na | 24447 | -0.082 | -0.3475 | No |
| 70 | EDNRB | na | 24896 | -0.089 | -0.3550 | No |
| 71 | WNT5A | na | 25202 | -0.094 | -0.3580 | No |
| 72 | FZD10 | na | 25265 | -0.095 | -0.3539 | No |
| 73 | PRKACB | na | 26457 | -0.116 | -0.3812 | No |
| 74 | CALM1 | na | 26557 | -0.117 | -0.3768 | No |
| 75 | DVL2 | na | 26784 | -0.122 | -0.3758 | No |
| 76 | FZD1 | na | 27779 | -0.143 | -0.3957 | No |
| 77 | KIT | na | 28047 | -0.150 | -0.3941 | No |
| 78 | KITLG | na | 28228 | -0.154 | -0.3897 | No |
| 79 | GNAI1 | na | 28406 | -0.159 | -0.3850 | No |
| 80 | WNT8B | na | 29287 | -0.184 | -0.3990 | No |
| 81 | WNT16 | na | 30080 | -0.209 | -0.4090 | No |
| 82 | FZD4 | na | 30972 | -0.241 | -0.4198 | Yes |
| 83 | FZD3 | na | 31028 | -0.243 | -0.4064 | Yes |
| 84 | PLCB2 | na | 31149 | -0.248 | -0.3945 | Yes |
| 85 | PRKX | na | 31362 | -0.257 | -0.3847 | Yes |
| 86 | FZD8 | na | 31536 | -0.265 | -0.3733 | Yes |
| 87 | WNT6 | na | 31862 | -0.283 | -0.3652 | Yes |
| 88 | ASIP | na | 32224 | -0.304 | -0.3568 | Yes |
| 89 | PLCB1 | na | 32371 | -0.315 | -0.3416 | Yes |
| 90 | DCT | na | 32728 | -0.339 | -0.3309 | Yes |
| 91 | POMC | na | 32781 | -0.344 | -0.3111 | Yes |
| 92 | PRKACG | na | 33174 | -0.374 | -0.2993 | Yes |
| 93 | FZD7 | na | 33284 | -0.383 | -0.2787 | Yes |
| 94 | FZD2 | na | 33686 | -0.431 | -0.2637 | Yes |
| 95 | LEF1 | na | 34024 | -0.487 | -0.2433 | Yes |
| 96 | PRKCB | na | 34175 | -0.520 | -0.2155 | Yes |
| 97 | WNT5B | na | 34219 | -0.533 | -0.1838 | Yes |
| 98 | MITF | na | 34414 | -0.606 | -0.1519 | Yes |
| 99 | WNT11 | na | 34469 | -0.631 | -0.1144 | Yes |
| 100 | TYRP1 | na | 34684 | -0.897 | -0.0651 | Yes |
| 101 | TYR | na | 34713 | -1.073 | 0.0004 | Yes |
Table: GSEA details [plain text format]

  

Fig 2: KEGG\_MELANOGENESIS      
 Blue-Pink O' Gram in the Space of the Analyzed GeneSet

  

Fig 3: KEGG\_MELANOGENESIS: Random ES distribution      
 Gene set null distribution of ES for **KEGG\_MELANOGENESIS**
